# Sensory integration combined with interoceptive interventions for functional urinary incontinence in children: a case report

**DOI:** 10.3389/fresc.2025.1599599

**Published:** 2025-07-08

**Authors:** Lingshan Ma, Yuanxun Zhang, Songjun Yao, Shuang He, Jie Li

**Affiliations:** Department of Pediatric Rehabilitation, People’s Hospital of Guangxi Zhuang Autonomous Region, Nanning, China

**Keywords:** case report, urinary incontinence, nocturnal enuresis, sensory integration, interoception

## Abstract

**Background:**

Functional urinary incontinence is prevalent among children and affects their daily life, psychology, and behavior. Although some behavioral therapies have been reported before, there is still no consensus on the treatment plan for functional urinary incontinence in children.

**Case information:**

This case describes an 8-year-old girl presenting with urgency, urine leakage and frequent nocturnal enuresis. An 8-week sensory integration therapy combined with interoceptive training was implemented, followed by follow-up visits at 4 and 8 weeks after the intervention. Her urinary urgency and leakage symptoms gradually decreased over the 8 weeks of treatment and resolved completely by week 12. However, nocturnal enuresis persisted, suggesting the limited efficacy of sensory-based interventions in non-awake states.

**Conclusions:**

The therapeutic effect of this case study indicates that interoceptive-sensory integration training has a positive effect on impaired bladder perception and voiding control during wakefulness and provides a new perspective for the evaluation and treatment of functional urinary incontinence. However, the improvement of enuresis by sensory enhancement still needs further research.

## Introduction

Functional urinary incontinence (FUI) in children refers to involuntary leakage of urine that is not caused by anatomical or neurological pathologies. Risk factors for urinary incontinence (UI) have been identified and can be categorized into psychological, behavioral, genetic, and social factors. The International Children's Continence Society (ICCS) divides FUI into daytime urinary incontinence and nocturnal enuresis, which can exist separately or together. Subtypes of daytime urinary incontinence include overactive bladder, dysfunctional voiding, underactive bladder, voiding postponement, and stress incontinence (1). Nocturnal enuresis is subclassified into non-monosymptomatic and monosymptomatic forms based on the presence or absence of daytime lower urinary tract symptoms (LUTS). Approximately 5%–10% of 7-year-old children experience frequent bedwetting, with a familial predisposition to this condition (2). Nocturnal polyuria, nocturnal detrusor overactivity, and high arousal thresholds are currently recognized pathogenic mechanisms (3). There are significant differences in global sensory processing in school-aged children with UI, especially in seeks sensation/under responsive, tactile sensitivity, and auditory sensitivity (4).

The sensory integration theory proposed by American occupational therapist A. Jean Ayres, PhD, in the 1970s has been widely applied to people with difficulties in sensory processing. She suggested that when the brain fails to integrate sensory information from the body's internal (proprioception, vestibular, and interoception) and external environments (auditory, visual, taste, smell, and touch), individuals may experience behavioral problems, learning difficulties, and emotional problems (5). Here, we report a case of FUI successfully managed with sensory integration techniques and interoceptive training.

## Case presentation

An 8-year-old girl presented to the pediatric department in August 2024 with urgency, urine leakage and frequent nocturnal enuresis. During the day, she frequently felt the urgent urge to urinate and sometimes wet her underwear before reaching the toilet, as well as experiencing occasional urine leakage during exercise or while passing gas. This girl's mother had to set 4 alarm clocks every night to remind the girl to get up and urinate. Once she turned off any alarm clock, the girl wet the bed. She reported that she went to the pediatric department of a public hospital for the first time due to the above symptoms in 2021 at the age of 5 years. The ultrasound examination of the bladder revealed that its size was approximately 9.0 × 5.5 × 6.4 cm, with a capacity of about 165 ml when filled. After urination, the bladder measured roughly 4.1 × 2.2 × 3.0 cm and had a capacity of around 14 ml. She was treated with oral Chinese medicine first, but the Chinese medicine treatment did not cure the girl. Therefore, the traditional Chinese medicine treatment was stopped and changed to take desmopressin tablets 0.2 mg every night before going to bed. When treated with desmopressin tablets, the symptoms of urgency and urine leakage did not change significantly compared with before, but the frequency of enuresis decreased to 2–3 times per night. Later, she tried the enuresis alarm at another hospital, which sends a strong wake-up signal when activated by urine. The treatment was not used continuously because she could not be aroused by the signal and experienced bedwetting.

There was no unexplained weight loss, polydipsia, or hyperphagia. The physical examination revealed no abnormalities. Anteroposterior and lateral views of the lumbosacral spine by digital radiography (DR) demonstrated no pathological findings. Color Doppler ultrasound examinations of both kidneys, bladder, and ureters showed normal anatomical structures without detectable anomalies. Urinalysis results were within normal reference ranges. There was no urinary tract infection, and the urine specific gravity value was 1.015, which was within the normal reference range. The urine sugar test was negative. Family history showed that her uncle and cousin had similar symptoms as school-age children, and her uncle's symptoms were still present at age 15. She was diagnosed with functional incontinence and referred to our department for conservative treatment. Based on her symptoms and medical history, she was advised to use sensory integration methods along with endosensory augmentation techniques to enhance her awareness of the urge to urinate and improve her control over urination during waking hours.

## Intervention

For the next 8 weeks, the intervention protocol was developed by a CLASI-certified therapist and validated for fidelity by another CLASI-certified practitioner, adhering to the principles of Ayres Sensory Integration (ASI). The program aimed to enhance arousal levels during wakefulness through vestibular, tactile, and proprioceptive activities, combined with body scanning and bladder vibration to improve awareness of bladder sensations. Specific implementation involved dynamic adjustment of intervention intensity and frequency based on symptom progression across initial, intermediate, and final intervention phases (Table 1). Follow-ups were performed at 4 and 8 weeks after the outpatient intervention. A bladder diary was utilized to document urinary frequency, the volume of urine voided, and the sensation of the bladder. Bladder sensation was assessed using the International Consultation of Incontinence Questionnaire-bladder diary (ICIQ-bladder diary) (6), which described how the bladder felt when going to the toilet using 5 codes: (0) (absence of sensation with socially motivated voiding), (1) (normal desire to void), (2) (urgency that subsided before reaching the toilet), (3) (urgency without incontinence), and (4) (urgency with incontinence). The girl was told not to change her past eating habits during the intervention to eliminate the effect of diet on the outcome of the intervention. In addition, she took 0.2 mg of desmopressin tablets at bedtime every night during the intervention.

**Table 1:** Description of intervention protocol.

Week (s)	Intervention type	Intervention protocol	Duration and frequency
Week 1	Outpatient intervention	- Sensory integration activities to enhance arousal levels - Supine body scanning - Finger percussion over bladder projection area - Limb cyclic compression	5 sessions/week
			30 min/session
	Home intervention	- Body scanning - Bladder awareness exercises	3 times/day
Week 2	Outpatient intervention	- Sensory integration activities to enhance arousal levels - Diaphragmatic breathing-integrated supine scanning - Bladder percussion - Limb cyclic compression	5 sessions/week
			30 min/session
	Home intervention	- Body scanning - Bladder awareness exercises - 10 slow deep squats	3 times/day
Weeks 3–8	Outpatient intervention	- Sensory integration activities to enhance arousal levels - Non-supine body scanning - Bladder vibration - Breath-holding abdominal compression post-inspiration - Supine hand-to-foot pass	2 sessions/week
			30 min/session
	Home intervention	- Body scanning - Bladder awareness exercises - 15-second horse stance holds	3 times/day
Weeks 9–16	Home intervention	- Body scanning - Bladder awareness exercises - Activity with semi-distended bladder	Ad libitum

## Outcomes

**Figure 1:**
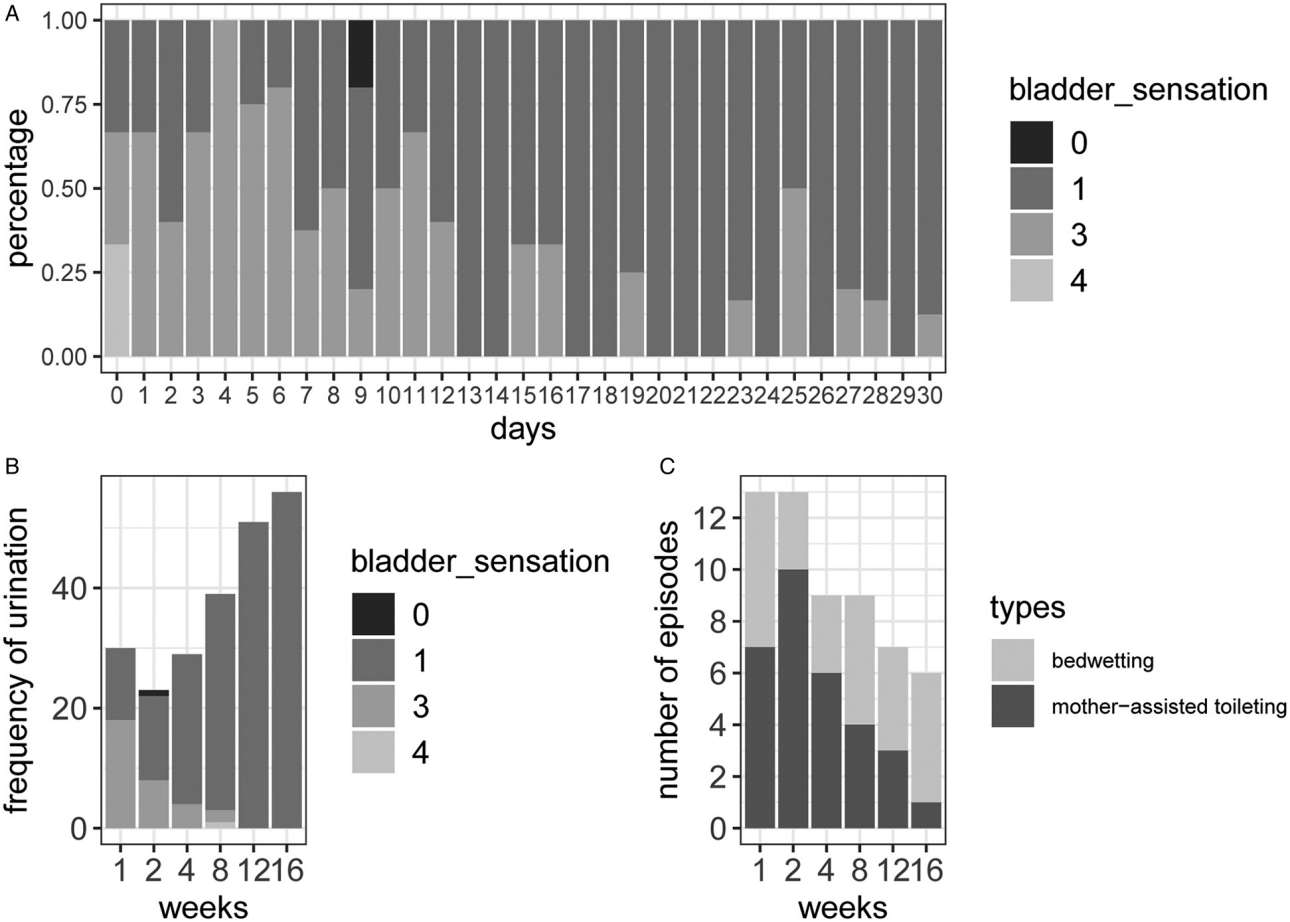
Dynamic changes in bladder sensation and nocturnal voiding patterns across intervention and follow-up phases. **(A)** Daily self-reported bladder sensation scores when going to the toilet over 30 consecutive days of intervention. **(B)** Weekly self-reported bladder sensation scores during intervention and follow-up. **(C)** Weekly nocturnal enuresis frequency and mother-assisted scheduled voiding episodes during intervention and follow-up. Sensation codes: 0 (absence of sensation with socially motivated voiding), (1) (normal desire to void), (2) (urgency that subsided before reaching the toilet), (3) (urgency without incontinence), and (4) (urgency with incontinence).

Bladder sensation dynamics throughout the study are visualized in Figure 1. Figure 1A illustrates the daily changes in self-reported bladder sensation during toileting episodes from baseline (day 0) to intervention days (days 1–30). A progressive enhancement in bladder perception was observed, with bladder sensation codes transitioning from predominantly urgency (codes 3–4) at baseline to mainly normal perception (code 1) by day 30. Longitudinal outcomes across the intervention and follow-up phases are presented in Figure 1B. Data collected at weeks 1, 2, 4, 8, 12 and 16 demonstrated three critical phases: (1) an early adaptive period (weeks 1–2), (2) a stabilization phase (weeks 3–8), and (3) follow-ups at 4 and 8 weeks after the outpatient intervention (weeks 12 and 16). Importantly, follow-up assessments at weeks 12 and 16 confirmed maintenance of therapeutic gains, suggesting lasting recalibration of interoceptive processing. Her urinary urgency and leakage symptoms gradually decreased over the 8 weeks of treatment and resolved completely by week 12. The total number of times she wet the bed and was woken up by her mother to go to the toilet was gradually reduced with the intervention (Figure 1C). The percentage of the total combined number of times per night that she wet the bed and was awakened by her mother to get up to urinate was greater than 1 was 85.71%, 57.14%, 28.57%, 28.57%, 0%, and 0% at weeks 1, 2, 4, 8, 12, and 16, in that order. In weeks 12 and 16, she either wet the bed once a night or was woken up by her mother to go to the toilet once a night. She and her parents were satisfied with the results, although nocturnal enuresis persisted.

## Discussion

There are various clinical subtypes of urinary incontinence based on their causes and symptoms. According to the International Continence Society (ICS), the most common types of UI are stress UI, urge UI, and mixed UI, and their first-line treatment is lifestyle and behavioral therapy, primarily consisting of pelvic floor muscle training (PFMT) (7). The girl in this case exhibited mixed urinary incontinence, characterized by urgency-associated leakage and stress-induced leakage during physical activities or farting. Although drug interventions have been used in the past, their efficacy has not met the expectations of this patient. It was hypothesized that her urinary incontinence was related to the brain's perception of and response to the bladder, from the fact that she “did not feel her bladder filling up and urinating involuntarily during sleep,” and that she “did not usually feel her bladder filling up during the day but when she did she had a strong urge to urinate and was difficult to control.” The pathophysiology of urge incontinence has been reported to be related to uninhibited detrusor muscle contractions, intrinsic detrusor muscle activation, impaired sensory pathways at the bladder, spinal cord, and/or cortical levels (7). When the bladder-filling sensation is blunted or delayed, the patient may not be able to perceive the normal urge to urinate and only experience an urgent urge to urinate when the detrusor muscle contracts strongly. Therefore, we chose to intervene with sensory integration technology and prioritized the treatment of visceral, tactile, and proprioceptive senses in the waking state to improve her bladder perception and urination control.

The first-line treatment for children with daytime urinary incontinence is urotherapy, which mainly includes education regarding diet, fluid intake and toilet position, urination pattern instructions, and bladder training (8). Besides, physical therapies such as PFMT, abdominal muscle training, core stability training, and biofeedback have also been shown to treat daytime functional urinary incontinence in children (9). Behavioral interventions and enuresis alarms are often used to treat nocturnal enuresis. Compared with no treatment or control, 65% of nocturnal enuresis children aged 5–16 years achieved a complete response after using standard alarm training (10). Sensory integration techniques can be applied to primary nocturnal enuresis, but there are few studies on daytime incontinence. It was reported that when the bladder was filled with more than strong desire to void volumes, the interoceptive network, including the insula, anterior cingulate cortex (ACC) and mid-cingulate cortex (MCC), was significantly activated in urgency urinary incontinence patients compared to controls (11). The signal of bladder dilation is uploaded to the insula and interoceptive afferent cortex via the thalamus, thus forming the sensation of the bladder and the desire to urinate (12). Therefore, it can be inferred that interoceptive training can enhance bladder signal detection and improve cortical-brainstem regulation.

Our research has some limitations. Improvement in urinary incontinence symptoms was only observed in a single case and is not universal. We conducted two follow-ups at 4 and 8 weeks after the outpatient intervention. Although the short-term effect is positive, longer follow-up is needed in subsequent studies to assess the persistence of the impact comprehensively. The findings of this study focused on a patient with mixed incontinence, and the role of other subtypes of incontinence is unknown. Therefore, further research is needed to explore the intervention effect of interoceptive-sensory integration training on different subtypes of urinary incontinence.

## Conclusion

Interoceptive, tactile, and proprioceptive enhancement and praxis training have positive effects on individuals with impaired perception of urination and control of urination, suggesting that sensory processing plays an important role in the pathophysiology of functional urinary incontinence. Therefore, it can be inferred that sensory integration technology and interoceptive regulation technology can improve daytime urinary incontinence, but further research is needed to improve urinary incontinence in non-awakened states.

## Data Availability

The original contributions presented in the study are included in the article/Supplementary Material, further inquiries can be directed to the corresponding author.

## References

[1] Austin PF Bauer SB Bower W Chase J Franco I Hoebeke P The standardization of terminology of lower urinary tract function in children and adolescents: update report from the standardization committee of the international children’s continence society. Neurourol Urodyn. (2016) 35:471–81. 10.1002/nau.22751 25772695

[2] Nevéus T Fonseca E Franco I Kawauchi A Kovacevic L Nieuwhof-Leppink A Management and treatment of nocturnal enuresis-an updated standardization document from the international children’s continence society. J Pediatr Urol. (2020) 16:10–9. 10.1016/j.jpurol.2019.12.020 32278657

[3] Nevéus T. Nocturnal enuresis-theoretic background and practical guidelines. Pediatr Nephrol. (2011) 26:1207–14. 10.1007/s00467-011-1762-8 21267599 PMC3119803

[4] Cupelli ET Escallier L Galambos N Xiang S Franco I. Sensory processing differences and urinary incontinence in school-aged children. J Pediatr Urol. (2014) 10:880–5. 10.1016/j.jpurol.2014.01.002 24636484

[5] Miller LJ Anzalone ME Lane SJ Cermak SA Osten ET. Concept evolution in sensory integration: a proposed nosology for diagnosis. Am J Occup Ther. (2007) 61:135–40. 10.5014/ajot.61.2.135 17436834

[6] Bright E Cotterill N Drake M Abrams P. Developing and validating the international consultation on incontinence questionnaire bladder diary. Eur Urol. (2014) 66:294–300. 10.1016/j.eururo.2014.02.057 24647230

[7] Vaughan CP Markland AD. Urinary incontinence in women. Ann Intern Med. (2020) 172:Itc17–itc32. 10.7326/aitc202002040 32016335

[8] Schäfer SK Niemczyk J von Gontard A Pospeschill M Becker N Equit M. Standard urotherapy as first-line intervention for daytime incontinence: a meta-analysis. Eur Child Adolesc Psychiatry. (2018) 27:949–64. 10.1007/s00787-017-1051-6 28948380

[9] Buckley BS Sanders CD Spineli L Deng Q Kwong JS. Conservative interventions for treating functional daytime urinary incontinence in children. Cochrane Database Syst Rev. (2019) 9:Cd012367. 10.1002/14651858.CD012367.pub2 31532563 PMC6749940

[10] Caldwell PH Codarini M Stewart F Hahn D Sureshkumar P. Alarm interventions for nocturnal enuresis in children. Cochrane Database Syst Rev. (2020) 5:Cd002911. 10.1002/14651858.CD002911.pub3 32364251 PMC7197139

[11] Ketai LH Komesu YM Dodd AB Rogers RG Ling JM Mayer AR. Urgency urinary incontinence and the interoceptive network: a functional magnetic resonance imaging study. Am J Obstet Gynecol. (2016) 215:449.e1–.e17. 10.1016/j.ajog.2016.04.056 27173081 PMC5045785

[12] Pang D Gao Y Liao L. Functional brain imaging and central control of the bladder in health and disease. Front Physiol. (2022) 13:914963. 10.3389/fphys.2022.914963 36035497 PMC9411744

